# Atmospheric Sampling of Persistent Organic Pollutants: Needs, Applications and Advances in Passive Air Sampling Techniques

**DOI:** 10.1100/tsw.2001.255

**Published:** 2001-10-16

**Authors:** Wendy A. Ockenden, Foday M. Jaward, Kevin C. Jones

**Affiliations:** Environmental Science Department, Lancaster University, Lancaster, LA1 4YQ, UK

**Keywords:** organic pollutants, air pollution, passive sampling, sampling, SPMD, SPME

## Abstract

There are numerous potential applications for validated passive sampling techniques to measure persistent organic pollutants (POPs) in the atmosphere, but such techniques are still in their infancy. Potential uses include: monitoring to check for regulatory compliance and identification of potential sources; cheap/efficient reconnaissance surveying of the spatial distribution of POPs; and deployment in studies to investigate environmental processes affecting POP cycling. This article reviews and discusses the principles and needs of passive sampling methodologies. The timescales required for analytical purposes and for the scientific objectives of the study are critical in the choice and design of a passive sampler. Some techniques may operate over the timescales of hours/days, others over weeks/months/years. We distinguish between approaches based on "kinetic uptake" and "equilibrium partitioning". We highlight potentially useful techniques and discuss their potential advantages, disadvantages, and research requirements, drawing attention to the urgent need for detailed studies of sampler performance and calibration.

## INTRODUCTION

There is a need to identify and quantify organic micropollutants in the atmosphere. Many compounds in air can transfer to humans and wildlife and have been linked to adverse health effects, even at low concentrations[1]. The severity and type of problems are dependent on the concentration and mixture of compounds present in the atmosphere[2]. Increased legislation and adoption of air quality standards require monitoring for compliance purposes. Monitoring is also often required in spatial studies, for example, around (suspected) point sources. The atmosphere is critical to the redistribution of organic micropollutants locally, regionally, and globally, with detection in remote locations far from use/source often being reported[3]. Air concentration data, ideally sampled at many sites simultaneously, are therefore also needed to construct and test/validate national or regional-scale chemical fate and behaviour models.

## PERSISTENT ORGANIC POLLUTANTS (POPs)

Persistent organic pollutants (POPs) are organic micropollutants of current research interest in the atmosphere. Many compound classes with a chain, branched chain, ring, or multiring backbone fall under this broad classification (Table 1). Their physical and chemical properties result in their resistance to photolytic, biological, or chemical degradation. They are usually lipophilic and can therefore accumulate through food chains. POPs can either be produced intentionally (e.g., organochlorine [OC] pesticides and polychlorinated biphenyls [PCBs]) or accidentally as by-products of, for example, chemical manufacture or incineration processes (e.g., polychlorinated dibenzo[*p*]-dioxins and furans [PCDD/Fs] and polynuclear aromatic hydrocarbons [PAHs]). POPs have been detected in the atmosphere of regions far removed from possible sources — e.g., the Arctic and Antarctic[4,5,6,7,8,9,10,11,12]. The concentrations and mixtures of POPs in the atmosphere vary widely in space, time, and with compound class. Individual PAH compounds are typically present in the rural/urban air of industrialised countries in the 1–10 ng $m^{-3}$ range, PCB congeners in the 1–10 pg $m^{-3}$ range, with PCDD/Fs typically two orders of magnitude lower again[13,14,15,16]. The sensitivity of modern bench-top gas chromatography/mass spectrometry (GC–MS) systems typically is in the range of a few 10s pg on column; hence, "large" air volumes — of the order of 10s to several 100s $m^3$ need to be sampled in order to reliably detect many of the POPs present in air.

**TABLE 1**
**Examples of Compounds that are Classified as Persistent Organic Pollutants (POPs)**

| Compound/Class | Use/Source |
|---|---|
| Polychlorinated biphenyls (PCBs) | Stable fluids, used in transformers, capacitors etc. |
| Polychlorinated dibenzo-*p*-dioxins and furans (PCDD/Fs) | Accidentally formed in combustion processes and impurities in some chlorinated chemicals |
| Polybrominated diphenyl ethers (PBDEs) | Flame retardant chemicals |
| Organochlorine (OC) pesticides | Agrochemicals |
| Polynuclear aromatic hydrocarbons (PAHs) | Incomplete combustion by-products, released from burning coal/wood/ petrol/ waste etc. |

Depending on ambient conditions, POPs can partition between the vapour and particulate phases of the atmosphere. This distribution will depend on the compound’s physical-chemical properties, the temperature, and the amount and nature of particulate matter (see Fig. 1). Relatively volatile species, such as the OC pesticide hexachlorobenzene, will be principally gaseous under most ambient temperatures worldwide. Less volatile species, such as octachlorinated dioxin, will be primarily particle associated, even in warm tropical locations.

### Active Sampling

Due to their low atmospheric concentrations, a large volume of air needs to be sampled to facilitate detection of POPs. The most common sampling methods therefore use a pump to actively draw air through a sampling train. This generally consists of a filter, either glass fibre or quartz, to retain nominally ascribed particulate material, and then an absorbent, such as polyurethane foam (PUF), Tenax®, or XAD resins[13,14,15,16,17], to retain the vapour-phase components (Fig. 2A). Alternatively, diffusion denuders can be used[18]. These sample the

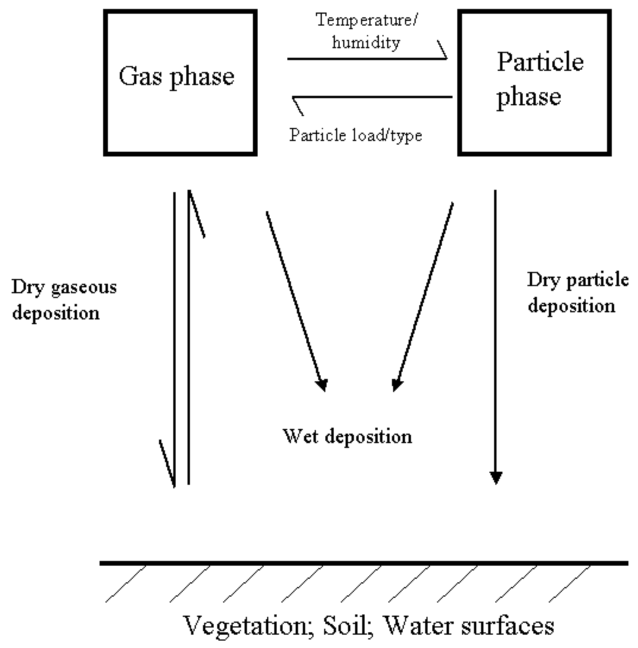

**FIGURE 1**. POPs in the atmosphere and their interactions with surfaces.

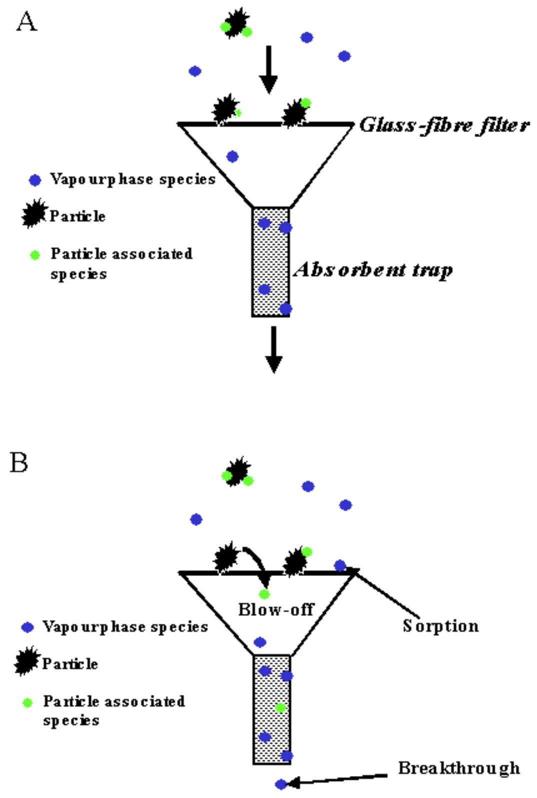

**FIGURE 2.** (A) Schematic of active air sampling. Air is drawn through the filter and adsorbent trap by a motor. Particle-associated species are retained by the filter, and vapour phase–associated species by the adsorbent trap. (B) Artifacts of active air sampling include blow off (species that were associated with particles, desorb, and are retained by adsorbent trap rather than the filter), breakthrough (vapour-phase species breakthrough from the adsorbent trap), and sorption of vapour-phase species to the filter.

vapour-phase components before sampling the remaining particulate species, or if the analytes are reactive, then an impinger system is required. By their very nature, pumped procedures disturb the air system, and as a consequence erroneous results may occur where, for example, vapour-phase species can be falsely ascribed as particle-associated species and vice versa (Fig. 2B). The major drawbacks with active sampling are that the equipment is generally expensive, skilled/trained operators are required to be on hand to run the equipment, and an electrical supply is needed to run the pump system. The latter, in particular, prevents sampling in many locations.

In recent years, work has been carried out using alternative "passive" sampling techniques — i.e., sampling without the need for electrical power. These sample atmospheric species by means of gaseous diffusion and sorption and/or permeation. Their main advantages are that they are generally cheaper and easier to use than active sampling methods. Because they do not require electrical power, more widespread deployment is possible. As legislation requires evermore stringent monitoring of POPs in air, the need for robust, validated passive air sampling methods will become even greater. This paper reviews passive sampling methods for POPs and then makes suggestions for developments/improvements in future.

## Passive Sampling

When sampling air passively, it is important to consider what is it that you want to measure, and what the sampler will "see". The vapour and particulate phase constituents in air can both be important, but will be supplied to the sampler by different deposition processes. These issues are illustrated in Fig. 1.

## Vapour Phase Sampling

### *Some Introductory Comments on the Principles and Objectives*

POPs undergo dynamic exchange between water, soil and vegetation, and the atmosphere (Fig. 1). These processes are important in controlling the entry of POPs into food chains, influencing their long-range atmospheric transport (LRAT) potential, and therefore controlling their global cycling and redistribution. It is therefore particularly important to measure/monitor the vapour-phase species in the atmosphere.

Fig. 3A shows how the POP concentration of a surface (e.g., a passive sampler) that comes into contact with vapour phase species in the atmosphere will respond. For a given, fixed gas phase concentration and temperature there is:

1. Initial linear uptake of compound onto the surface;
2. A curvilinear portion of the uptake curve as equilibrium partitioning is approached;
3. Equilibrium between the concentration at the surface and in the gas phase of the air.

It should be noted that the eventual mass of compound held by the surface when it is at equilibrium with the air depends on the temperature, the type of surface being considered (i.e., inherent properties of the surface material to sorb/retain the POP), and the physicochemical properties of the POP. For example, a lipid surface such as octanol would retain a greater mass of a particular POP than a surface of water. Equally, an octanol surface would retain a greater mass of POP with a high octanol–air partition coefficient ($K_{OA}$) (e.g., PCB-180) than it would a POP with a low $K_{OA}$ value (e.g., PCB-8). If the POP of interest becomes absorbed into the sampler, diffusing below the surface, it will have a greater capacity for "sampling" the air. It then becomes appropriate to consider an additional "complication" to the uptake curve shown in Fig. 3A, namely that the loading of POP on the sampler surface is influenced by the "size" or capacity of the subsurface compartment (Fig. 3B). The rates of two processes then become important — the

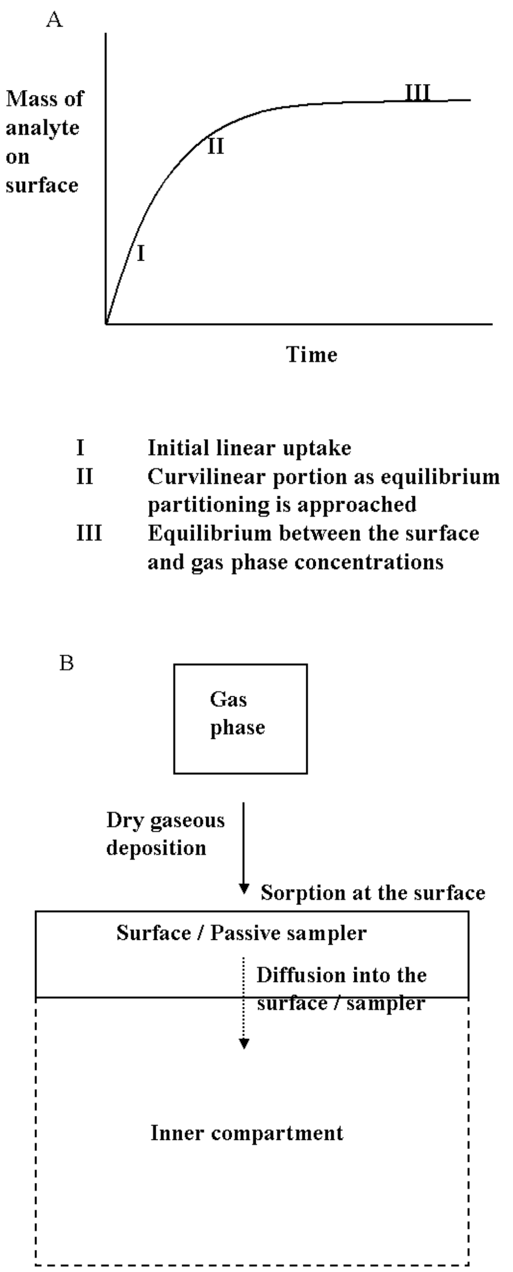

**FIGURE 3.** Schematic of the uptake of gas phase compounds by a surface/passive sampler.

rate at which gas-phase POP is supplied from the air to the sampler surface *and* the rate at which POP on the sampler surface diffuses into the subsurface compartment(s). As Fig. 4 shows, the rate of supply to the surface could potentially be influenced by transport through the bulk atmosphere (influenced by wind speed) or the rate of movement through the stagnant boundary layer (which is in turn influenced by the boundary layer thickness). The boundary layer thickness will itself be influenced by wind speed.

The equilibrium concentration of the surface/sampler is a function of the compound air–surface partition coefficient and the temperature (and possibly humidity). The total mass of compound retained by the sampler is influenced by the capacity of the material for the POP of interest and

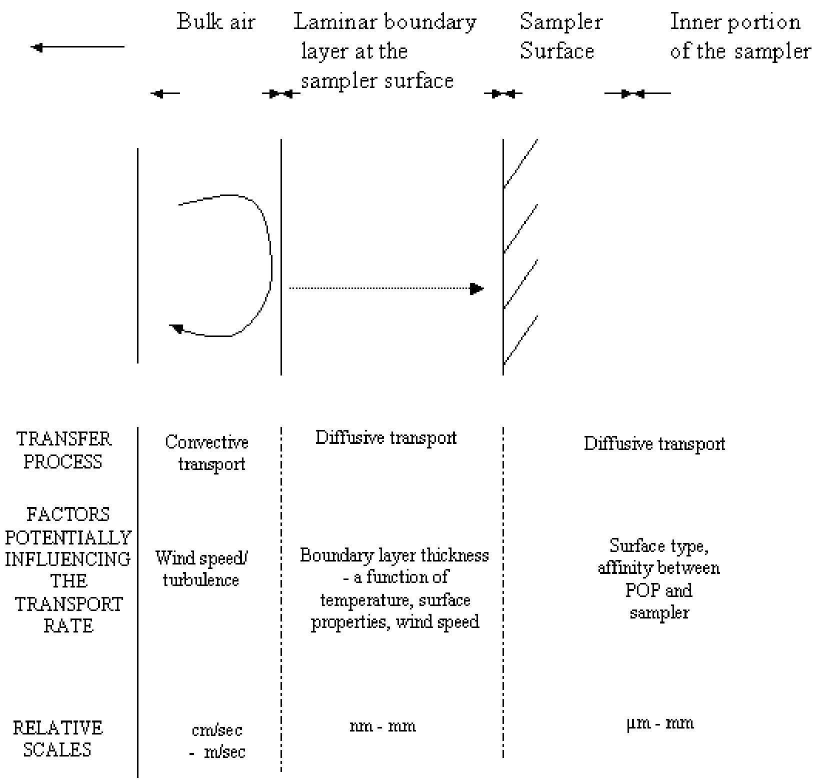

**FIGURE 4.** More detailed conceptual consideration of gas-phase POP transfer to a sampler.

the surface area and thickness of the sampler (i.e., the total sorptive capacity of the sampler). Clearly these are all factors that can be varied to optimise the choice/design of sampler type. It might be appropriate or necessary to vary the sampler type and configuration, depending on the compound(s) of interest, the sampling/deployment time, and the sensitivity of the analytical instrumentation available.

From Fig. 3A, it becomes clear that there are two strategies that can be exploited with passive sampling. These are to sample during the linear/curvilinear uptake portion of the curve (*kinetic sampling*) or once equilibrium conditions have been attained (*equilibrium sampling*). With the former, compound-specific uptake rates would need to be determined, with the influence of temperature, wind speed, and other environmental variables fully characterised and quantifiable. With the latter, the sampler–gas phase partition coefficient would need to be known, together with knowledge of its temperature dependency. The time to equilibrium would also need to be known — i.e., samplers would need to be exposed to the air for long enough to ensure that they had attained equilibrium. The time to reach equilibrium varies between compounds and increases with the sampler–gas phase partition coefficient.

The issue of sampling time is important, and obviously depends on the objectives of a given study. Average (time-integrated) air concentrations over weeks or months may be useful for monitoring and reconnaissance work. Monitoring of accidental releases may require samplers that respond over shorter times — perhaps hours or days. Monitoring for occupational exposure would require samplers that respond in hours — through the working day. Studies of air-surface exchange fluxes, to better understand processes, would ideally be performed on a timescale of hours or even minutes. As noted above, the time to attain equilibrium will be a function of the capacity of the sampler and could be varied by maintaining flexibility in design. Where the need is for equilibrium-based short-term sampling, the capacity of the sampler would have to be very small, which may result in a trade-off with analytical sensitivity. It is unlikely that a single compound could be sampled by the same techniques to suit all study requirements, or that a single sampler type would be suitable for all POP compounds in the same study.

## Diffusion Tubes

A well-established way of sampling relatively abundant vapour-phase compounds from air is using diffusion tubes. These consist of a solid-phase absorbent, the sampling face of which may or may not be protected by some form of membrane. Sorbents such as Tenax®, Carbopack®, and charcoal have been used[19,20,21], with the choice of sorbent being analyte dependent. Uptake can be described using Fick's first law of diffusion (the mass transfer rate of gas during diffusion is directly proportional to the diffusivity of the gas in the air, the concentration gradient, and the diffusion path cross-sectional area)[22]. Knowledge of the diffusivity constant of an analyte allows calculation of the atmospheric concentration. Diffusion tubes have been successfully used for volatile organic compounds (VOCs), such as benzene and hydrocarbons[23,24]. However, POPs are generally at orders of magnitude lower concentrations in the atmosphere, so diffusion tubes are unable to sample sufficient analyte for reliable detection.

The main techniques that have been used (or show promise) to date for direct passive sampling of gaseous POPs are semipermeable membrane devices (SPMDs)[25,26,27,28,29,30,31,32] and solid-phase microextraction (SPME)[33,34]. Other techniques that have been used are glycerol-covered glass plates, Teflon surfaces, and PUF samplers[35,36,37]. Atmospheric concentrations have also been inferred from analyte concentrations in other matrices, e.g., butter, pine needles, and peat[38,39,40]. These approaches will now be discussed in some detail.

## Semipermeable Membrane Devices (SPMDs)

### *Introductory Remarks*

SPMDs consist of a sequestering solvent system enclosed within a semipermeable membrane. Analytes permeate through the membrane into the solvent, where they concentrate and are retained. Solvent systems that have been used include hexane and octanol. The major problem with these solvents is their volatility, which limits exposure times. Recent advances have been made using the synthetic lipid triolein (1,2,3-tri[cis-9-octadecenoyl]glycerol) as the sequestering solvent. Triolein-containing SPMDs were first designed by the U.S. Geological Survey (USGS) for water sampling of POPs[41,42]. After the discovery of high concentrations of analytes in blanks, it was realised that these samplers might be suitable as passive air samplers[25]. Much work has been carried out on these samplers, with uptake and sampling rates being obtained for a suite of POPs, including PCBs, PCDD/Fs, and PAHs[28,32]. The majority of this work has been carried out using standard, commercially available triolein-containing SPMDs. These are made of low-density 75-µm polyethylene membrane (80–90 cm in length) containing a thin film of triolein (1 ml per sampler). The capacity of triolein for POPs is large, allowing detection of ultratrace contaminants as long as exposure times are long enough.

Uptake by the SPMD can be envisaged as the three-step process discussed earlier and shown in Fig. 3A. Knowledge of whether uptake remains in the kinetic region (linear or curvilinear) or whether the SPMD and air are at equilibrium allows estimation of atmospheric concentrations. The triolein gives SPMDs a large storage capacity for POPs, so the "uptake phase" with these samplers is "long". However, POPs possess a huge range of properties, with orders of magnitude differences in partition coefficients and, therefore, in sampling times. The log octanol–air partition coefficient ($K_{OA}$) values for POPs range from ca. 5–6 for "light" species (e.g., naphthalene; lower chlorinated PCBs; HCHs) to ca. 11–12 for "heavy" species (e.g., benzo[a]pyrene; octachlorinated dioxin). Time to reach air-sampler equilibrium therefore ranges between tens of days to tens of years, depending on the POP[28,31,32].

## Comments on the Principles

If the air and the SPMD are at equilibrium, the mean atmospheric concentration ($C_{AIR}$, mass per unit volume) during deployment can be estimated from:

$$C_{AIR} = C_{SPMD} / K_{SPMD\text{-}AIR} \quad (1)$$

where $C_{SPMD}$ is the concentration of analyte sequestered by the SPMD (mass per unit volume lipid), and $K_{SPMD\text{-}AIR}$ is the SPMD–air partition coefficient corrected for mean temperature during deployment. To date, values for $K_{SPMD\text{-}AIR}$ are not available. It has been found that octanol–water partition coefficients ($K_{OW}$) are almost equal to triolein–water coefficients ($K_{TW}$)[43]. It seems justifiable, therefore, to assume that $K_{OA}$ will be closely related to triolein–air partition coefficients[28]. If it is assumed that it is just the triolein in the SPMD that has a capacity for analytes (i.e., the membrane capacity is ignored), then $K_{SPMD\text{-}AIR}$ in Eq. 1 can be replaced with $K_{OA}$. $K_{OA}$ has been measured or calculated for a large number of POPs, and information is available allowing correction for temperature[44,45]. A problem with this approach is that $K_{OW}$ and $K_{TW}$ are not exactly equal, and the difference between $K_{OW}$ and $K_{TW}$, and therefore, presumably, also between $K_{OA}$ and $K_{TA}$, increases with decreasing compound volatility/increasing $K_{OW}$[44]. In addition to the assumption that $K_{TA}$ and $K_{OA}$ are equal, a further drawback with this approach for estimating air concentration from the concentration sequestered by an SPMD is that it assumes that the SPMD membrane has no capacity for the POPs. This is undoubtedly wrong. SPMDs containing no lipid have been successfully used in water sampling of POPs, for example[46]. A more fundamental/logistical problem is the length of time required for the SPMD and the air to attain equilibrium. Work with PCBs, for example, has shown that for the less volatile congeners with five chlorines or more, it could be a matter of years/decades before the SPMD attains equilibrium[28]. This is obviously not a feasible deployment period for the majority of studies.

The most appropriate approach with SPMDs is therefore to keep exposure periods sufficiently short to be certain that SPMD uptake is linear. A calibration exercise is required to determine the sampling rate ($R_S$, volume air sampled per unit time) during the linear portion of uptake. During the calibration, atmospheric concentration ($C_{AIR}$, mass per unit volume) of a particular analyte should remain constant. $R_S$ can then be calculated from the uptake rate (U, gradient of a plot of mass analyte sequestered against time):

$$R_S = U / C_{AIR} \quad (2)$$

Mean air concentrations during a deployment of time, t, can then be calculated from the mass of analyte sequestered by the SPMD ($N_{SPMD}$):

$$C_{AIR} = N_{SPMD} / (R_S \times t) \quad (3)$$

Field-based calibration studies have been carried out for a range of POPs[28,32]. Drawbacks of these studies are that, because they were performed in the field, the atmospheric concentration, temperature, and wind speed varied during the exposure period. Nonetheless, they are the best data that are available to date. In addition, it should be noted that field-based studies have the advantages that they allow calibration at realistic environmental concentrations of analytes, and there is potential for a large number of analytes to be assessed simultaneously.

Sampling rates are compound dependent. It has been found that if analytes are associated entirely with the vapour phase of the air, then $R_S$ increases with increasing SPMD–air partition coefficients/decreasing volatility[28]. The shape of the molecule also appears to affect uptake rates, with decreasing molecular freedom resulting in decreased uptake rates, because of slower permeability through the membrane[28].

Obviously, this approach requires values for $R_S$ to be identical at different sites. Uptake rate is dependent on the temperature and possibly the wind speed[28,31]. The wind speed over the sampler could affect transfer of pollutants to the SPMD in two ways (Fig. 4). Very low wind speed could result in a laminar boundary layer at the SPMD surface (increase in the transfer coefficient and decrease in the sampling rate). Conversely, high wind speed would cause turbulence/eddies, causing a reduction in diffusion length and an increase in sampling rate. If airflow across the sampler can be buffered, the effect of differences in wind speed between sampling sites can be minimised[31]. Membrane permeability is also affected by humidity[47], which could also affect uptake rates of some compounds. Although no relationship has been elucidated, it is expected that the affect of humidity will be less than that of temperature or wind speed. It has been found that values for $R_S$ are greater in winter than in summer[28,31]. It has been postulated that this is due both to decreased temperatures (increased affinity of analyte for the lipid rather than the air) and increased wind speed in winter, although current thinking favours the former explanation.

Sampling rates calculated for PCBs at one site in the U.K. have been used in other studies to estimate atmospheric concentration, without correction of $R_S$ for temperature or wind speed (in all cases, however, SPMDs were deployed in screens to buffer differences in wind speed between sites)[29]. For example, sampling rates were calculated at 4 and 18ºC, and they were applied at a site where temperature ranged from –33 to +16ºC (mean = 6ºC). The calculated atmospheric concentrations were shown to be very similar to concentrations measured by an active sampling system during the exposure period[29].

## Research Needs and Applications

Ideally, work is required to correct $R_S$ for temperature and wind speed for different compounds. However, an alternative and very promising approach is to use performance-reference compounds (PRCs)[31]. These are compounds that are added to the interior of the SPMD prior to exposure. They should be isotopically labelled analytes or compounds that are not present in the matrix to be sampled. The physical-chemical properties of the PRCs used should be identical, or at least similar, to those of the target analytes. Loss rates of the PRC will be related to uptake rates of the target analytes, and sampling rate at a particular site during a particular exposure can be "corrected" — calculated from knowledge of the PRC loss rate. This approach has been successfully used in water sampling for many years[46,48], and a pilot study has suggested success with their use in passive air sampling[31].

As uptake kinetics cease to be linear, uptake can be described by:

$$N_{SPMD} = C_{AIR} \times V_{SPMD} \times K_{SPMD\text{-}AIR} \times [1 - \exp(-k_{UP} \times t)] \qquad (4)$$

where $k_{UP}$ is the uptake rate constant, and $V_{SPMD}$ is the volume of the SPMD[31].

In order for Eq. 4 to be used to estimate air concentration, $k_{UP}$ needs to be determined. $K_{SPMD\text{-}AIR}$ also needs to be known. As mentioned previously, in the absence of $K_{SPMD\text{-}AIR}$, $K_{OA}$ can be used. Again, a limitation of this approach is that it ignores the capacity of the membrane.

Despite the fact that more work is required to understand the kinetics of uptake, SPMDs show promise for use in passively monitoring POPs in air (see Table 2). At present, it is suggested that they can be used in a semiquantitative manner. Their use has been partially validated, and air concentrations calculated using sequestered concentrations have been shown to be very close to air concentrations derived from active sampling techniques. SPMDs show great potential for use in time-integrated studies, where deployment can be in the order of days, weeks, or months/years. SPMDs could currently be used in monitoring or reconnaissance studies, to highlight sites where accepted active sampling should be performed. However, until their use is fully validated they cannot be used in cases where litigation may result.

**TABLE 2**
**Summary of Potential Uses, Advantages, and Disadvantages of Passive Sampling Approaches for Vapour Phase POPs**

| Passive Sampler | Potential Uses | Advantages | Disadvantages/Remaining Questions |
|---|---|---|---|
| SPMD | Long-term, integrated kinetic sampling (days/months/years) | Large capacity; well-understood samplers from previous work in water; PRCs can potentially correct for different sampling rates between sites/studies | Complicated analytical clean-ups; uptake calibration studies still needed, together with clarification of key controlling variables; membrane and lipid may deteriorate over time following exposure to the environment, affecting sampling rates |
| SPME | Short-term studies, utilizing exposure times to reach equilibrium (hours to days) | No clean-up necessary; analytes introduced directly on column; capacity can be varied by altering coating thickness. | Environmental parameters and factors such as uptake kinetics and calibration are not fully understood; method still at the initial stage of development; uncertainties over particulate sampling |
| Vegetation | Depending on species used, can be suitable for both short- and long-term studies | Large capacity and covers major part of landmass | Only plants of same age and species are to be compared; no historical record available |
| PUF Disks | Long-term studies | Large capacity | Environmental parameters and factors such as uptake kinetics and calibration not fully understood; method still at the initial stage of development; uncertainties over particulate sampling |
| POGs | Short-term studies | Rapid air-sampler equilibration; flexibility, with the surface area and coating thickness being varied | See comments above for PUFs; prone to high blank contamination |

## Solid Phase Microextraction (SPME)

### *Introductory Remarks*

Like SPMDs, SPME was originally developed and applied to studying organic chemicals in aqueous systems and has had some limited applications to the passive sampling of relatively abundant compounds in air. In contrast to SPMDs, with their large capacity and sampling based on the kinetic phase of uptake (Fig. 3), SPMEs have a very small surface area and storage capacity for POPs. Hence, they equilibrate quickly, and the principle of their use is to allow exposure to be long enough that they reach equilibrium partitioning with the surrounding medium; this could be the dissolved phase in water or — in the applications considered here — the gas phase in air. This is "rapid" compared to SPMDs — likely of the order of hours/days for POPs in air. SPME therefore lends itself to different applications from SPMDs.

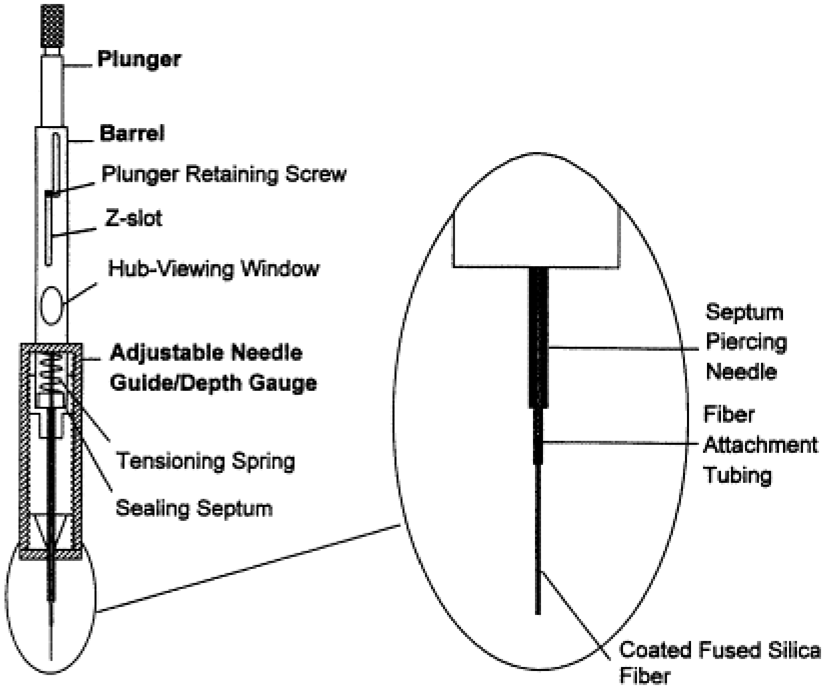

**FIGURE 5.** Schematics of SPME manual sampler.

Pawliszyn and Liu originally developed SPME in 1987[33]. The SPME device consists of a length of fused silica fibre coated with a liquid-polymer phase. In some cases, this is mixed with a solid adsorbent (Fig. 5). The fibre is attached to a stainless steel plunger sheathed by a protective needle. The plunger moves the fibre in and out of the needle to protect the coating. During sampling, the fibre is withdrawn from the needle of the device and exposed to the sample matrix. Analytes partition from the sample matrix onto the fibre, until equilibrium is reached[49,50,51,52,53,54,55,56,57,58,59]. The fibre is withdrawn into the needle of the SPME device and introduced directly into the injector port of a GC, by piercing the needle through a septum, and it is then extended into a hot GC injector, so that the analytes are then thermally desorbed onto the GC analytical column[49,50,51,60]. The direct transfer of analytes into the GC has tremendous analytical advantages, by eliminating the need for sample cleanup and preconcentration.

### *The Principles and Equilibrium Partitioning*

The principle of SPME is based on the equilibrium distribution partitioning of analytes between a sample matrix and the polymeric stationary phase coated on the silica fibre. At equilibrium, the amount of analyte absorbed by the coating will be directly proportional to the concentration of the analyte in the air and depends on the partition coefficient between the analyte and the fibre coating, $K_{SPME\text{-}AIR}$[51,53,60,61]. As in SPMD (Eq. 1), the mean atmospheric concentration ($C_{AIR}$, mass per unit volume) can be estimated from

$$C_{AIR} = C_{SPME} / K_{SPME\text{-}AIR} \qquad (5)$$

where $C_{SPME}$ is the concentration of analyte absorbed by the SPME (mass per unit volume of polymeric phase).

The polymeric coating can be either liquid or solid. A substantial difference exists between the performances of these two coatings. The analytes partition onto the extraction phase for liquid-coated fibres, where the molecules are solvated by the coating molecules. Solid-coated fibres, on the other hand, have a well-defined crystalline structure, which reduces the diffusion within the structure[50].

Different types of liquid coatings and coating thickness provide different absorption properties for different kinds of analytes. The performance of a fibre depends on the linearity, selectivity, interference, reproducibility, and sensitivity[58,62]. An increase in fibre coating thickness generally improves sensitivity but lengthens sampling time. A balance therefore has to be achieved, with fibre choice being analyte dependent. Polydimethylsiloxane (PDMS) coating is used to extract nonpolar compounds, such as PAHs, while polar compounds, such as phenols, are extracted by polyacrylate coating. In PDMS coating, for example, larger molecular weight or semivolatile compounds are more effectively extracted with a 30- or 7-µm film thickness, while a 100-µm film thickness is used to extract the lower molecular weight or volatile compounds. The 7-µm PDMS fibres with thinner films equilibrate faster, but they have a lower capacity and may not be useful for analysing semivolatile trace-level compounds[50,62].

The time needed for equilibrium to be established between the air and the fibre coating must be determined[49,50,51,58,63]. As noted above, equilibration time depends on the kinetics of the overall process of analyte uptake by the fibre — which may be controlled by the rate of analyte diffusion in the air and the partition coefficient of the analyte between the air matrix and fibre coating. These two factors are dependent on the nature of the compound, the fibre coating material, and the thickness of the fibre[64]. Equilibration time can be measured by exposing the fibre to a fixed gas-phase concentration for different lengths of time, until steady state is reached. Potential losses of analyte also need to be considered, by desorption, evaporation, photolysis, or microbial degradation[49,51].

### *Effects of Environmental Variables*

Temperature has two opposing effects on sampling efficiency. An increase in temperature enhances the diffusion of analytes through the atmospheric boundary layer towards the fibre. Conversely, an increase in temperature reduces the partition coefficient, resulting in a reduction in the equilibrium amount of analyte extracted[50,51]. Temperature also influences molecular mobility and, hence, diffusion into the SPME coating. The temperature dependency of equilibrium partitioning clearly needs to be established before routine use of SPME for passive air sampling can be considered[51,62]. Therefore, within a specified temperature range (ambient condition in the field), temperature does not have any significant effect on the amount of analytes extracted, since the temperature variation over a typical sampling event might not be large enough to be significant[62]. In the laboratory however, if the extraction rate is temperature dependent, then the highest temperature that still provides satisfactory sensitivity should be used[51].

Wind speed may influence the rate of supply of analyte to the fibre and, hence, time to equilibrium. Lord et al.[50] showed that very slow wind speeds (up to 5 cm/sec) affected mass transfer of VOCs from the bulk air to the fibre (Fig. 4). However, no further increase in the mass loading was observed for wind speed higher than 5 cm/sec, which they attributed to diffusion within the pores of the fibre then becoming the limiting factor.

Relative humidity greater than 90% was found to decrease analyte mass loading on the PDMS fibre by 10% and possibly change the hydrophobic nature of the PDMS (and other) fibre coatings[63]. A monolayer of water on the fibre coating will provide a hydrophilic surface, which may repel compounds with higher air–fibre partition coefficients, thereby affecting uptake. The formation of a water layer may also provide a medium for the partitioning of inorganic species including NOx and SOx and volatile biogenic organic acids, which will degrade the compounds being analysed and, even more importantly, degrade the fibre. Clearly the importance of these factors needs to be established.

## Calibration

Air sampling by SPME has been greatly hampered by the complexity of calibration as compared to liquid samples, where calibration is easily done by external or internal standards[53,65]. SPME GC–based analytical procedures (sorption of analytes on fibre, their desorption in a GC injector port, separation in a chromatographic column, detection, and quantitation) require careful calibration[66,67]. The principal drawback is the difficulty in preparing standards for gas sampling in a range of typical atmospheric concentrations. Furthermore, calibrations typically must be done at the same temperatures as those anticipated in the field. The following two criteria must be met before a gaseous mixture is considered to be a standard mixture[53]. The concentration of analytes of interest should be (1) known and (2) constant for a long period of time.

Various calibration methods have been suggested[63,68]. Martos et al.[63] developed a method for air sampling that is independent of temperature effects and does not require direct calibration for PDMS coatings. They related the GC response to absolute mass of a given analyte introduced into the GC system, by injection of standard liquid solutions using gaseous standard mixtures that contained analytes of interest at the appropriate concentrations. Here the sample and the standard gaseous mixtures are subjected to the same analytical operations.

Calibration has been achieved by the "static method"[69]. Here, an SPME fibre is exposed to a standard gaseous mixture generated in a glass bulb of known volume. The limitation of this method is that the sample volume is different from the volume of the glass bulb (volume of standard). Thus this method cannot work in field sampling, where the sample volume is unlimited, because the amount extracted by the fibre will be significantly larger. To solve this problem, the dynamic generation of standard gaseous mixture was developed[70]. In this method, there is an unlimited supply of standard gas mixtures. This approach eliminates the effect of sample volume, and if the system is allowed to reach equilibrium prior to the experiment, analyte losses are eliminated. This method can be used to calibrate the fibre for direct ambient air sampling.

## Partition Coefficients

Various methods have been used to determine the fibre coating–air partition coefficients for PDMS SPME fibres. Among them, the use of physicochemical data and chromatographic parameters has been shown to be an extremely reliable technique[51]. For example, the partition coefficient between a fibre coating and gaseous matrix can be estimated using isothermal GC retention times on a column with a stationary phase identical to the fibre coating material, since the partitioning processes in GC and SPME are similar, and there is a well-defined relationship between the partition coefficient and retention time[71]. The most useful method uses the Linear Temperature Program Retention Index (LTPRI) system that indexes a compound’s retention times in relation to the retention times of n-alkanes[72]. The LTPRI permits interpolation of the partition coefficient values from the plot of log $K_{SPME\text{-}AIR}$ vs. retention time. The LTPRI values for many compounds are available in literature; therefore this method allows $K_{SPME\text{-}AIR}$ estimation without experimentation. If the LTPRI value for a compound is not available from published sources, it can be determined from a GC run.

Partition coefficients can also be estimated using heat of vaporisation and activity coefficients from literature values and ascertained at temperatures for which a partition coefficient was not determined. This removes the restriction that calibration and sampling must be done at the same temperature[63]. However, if the partition coefficients for the target compounds are known, the above calibration methods might not be necessary[49].

The deployment of SPME in passive air sampling of POPs is still in its infancy and has yet to be proven. Table 2 summarises some potential advantages and disadvantages[49,50,52,53,54,58,60,61,65,73,74,75,76]. A number of practicalities may hinder progress, including: the fragile nature of the fibre; parameters like temperature, wind speed, humidity, sample volume, etc., and their effect on precision; interference from other compounds, which may also be taken up onto the fibre during field deployment; the low capacity of the fibre, which makes sample contamination a problem; and the calibration difficulties mentioned earlier.

## Inference Techniques

Vegetation sampling and analysis is one of the most common methods of making inferences about air concentrations[12,39,77,78,79]. It has been shown that the majority of a plant's aboveground POP burden originates from atmospheric deposition[80,81]. Therefore inference of air concentrations from vegetation concentrations (biomonitoring) offers attractive possibilities. Studies to date have used grass, pine needles, mosses, lichens, and other genre to attempt to estimate qualitative differences in air concentrations/profiles between sites. A major advantage of this method is that vegetation covers over 80% of the Earth's landmass[82]. Grass has been shown to respond rapidly to differences in atmospheric concentrations, making it suitable for short-term studies[83], while lichens, which are long lived, seem to have a much larger capacity for POPs, making them suitable for longer-term studies[12]. A disadvantage in the use of vegetation, however, is that it is essential that plants of the same age and species are compared between sites[12].

Rather than comparing concentrations in vegetation between sites in a semiqualitative manner, air-to-grass transfer coefficients have been calculated[83]. From these, if grass of a particular species is sampled, then it is possible to estimate the air concentration from the grass burden, as long as the age/exposure period of the plant is known. This method has recently been extended to the use of butter for estimating atmospheric concentration[38]. Air-to-grass transfer coefficients are known, as are grass-to-cow-to-butter/milk transfer coefficients. Therefore, by determining the POP concentration in a local butter or milk sample, the concentration in the grass that the cow ate can be estimated and, by extrapolation, that in the air that supplied the grass. However, it is known that factors other than air concentration will influence the POP concentration of butter — such as animal husbandry, stage in the lactation cycle, and the composition of feed supplements[84]. There are, therefore, potentially important confounding factors.

Many studies require historical air concentration data. Obviously this is more difficult to obtain than present-day concentrations. Methods that have been used to infer historic air concentrations include peat and ice/snow core samples[40,85,86,87]. Ombrotrophic peat bogs obtain their nutrients and, by inference, pollutants from the atmosphere. Peat cores can then be collected, sliced, dated, and analysed. Similarly, POPs are scavenged from the air by falling snow. Accumulation in a snowpack over time will therefore hold an historical record. Conductivity measurements can be used to age analysed slices[87]. The drawback of these approaches is that it must be assumed that there has been no postdepositional change in the pollutant burden. In ice work this has been shown to not be the case[86,87]. More volatile compounds are found to volatilise from the core, for example. In peat cores, leaching could also occur. In addition, smearing throughout the core could occur on sample collection, and errors may result from the aging of material.

## Other Techniques

A preliminary study has been carried out, using PUF disks as passive air samplers[37]. Uptake is seen to be linear over at least 2 months. Once sampling rates have been calculated for this type of sampler, it would be possible to estimate atmospheric concentrations, as for SPMDs (Eq. 3).

Initial work has also been carried out on the possibility of using polymer-coated glass slides (POGS) and thin layers of clean, uncontaminated soil[37]. These systems have a large surface area and a relatively low absorptive capacity. It has therefore been suggested that they will equilibrate with the atmosphere on a relatively short time scale, allowing calculation of atmospheric concentrations using a variableof Eq. 1 ($K_{TA}$ replaced with soil–air partition coefficients and polymer–air partition coefficients for soil and POGS, respectively). The thickness of the polymer coatings can be varied, to adjust their capacity, whilst they can also be developed for plates of different sizes[88]. Hence, they can be purpose-built to fit the requirements for certain compounds and detection systems. The analytical cleanup is also quite straightforward[88].

A recent study used tristearin-coated fibreglass sheets to passively sample POPs from the atmosphere[89]. A field calibration was carried out to assess uptake rates of these samplers. Other methods for semiqualitatively measuring POPs in air have included buckets of water, oil-coated glass sheets, and Teflon sheets[35,36,90]. None of these methods have gained great acceptance so far.

## Particulate Sampling

As mentioned, it is more important to assess concentrations of POPs in the vapour phase of the atmosphere than in the particulate phase. Particles and deposition can, however, confound results with all passive vapour samplers. For example, recent studies have shown that SPMDs can take up at least a small proportion of compound from particulates[30,32]. Compounds that occur almost entirely in the particle phase of the atmosphere were found to be sampled by SPMDs, although for some compounds, deployments of at least a year were required for reliable detection. It was also found that compounds that had been associated with particles adhering to the sticky surface of an SPMD could desorb from that particle and then permeate through the SPMD membrane, where subsequently they would be sequestered by the lipid[32]. However, it is not clear if this presents a problem to the routine use of SPMDs for air sampling. The most widely accepted processing procedures require that the surface of the sampler be cleaned, including organic solvent rinsing to remove any chemicals on the surface, before the sampler is processed to recover the sequestered analytes. In addition, the burden of particles which become associated with the SPMD surface during routine deployment may be minor in comparison to the much more efficient uptake of gas phase POPs.

Sampling of gaseous compounds relies on diffusion. Diffusion of atmospheric particles is much lower — the coefficient of diffusion is six orders of magnitude lower for a 0.3-μm diameter particle than for an oxygen molecule[91]. Capture by gravitational forces is possible for larger particles. However, in environmental monitoring, it is generally the smaller particles that are of concern.

Preliminary data are available for a passive sampler for urban particulate material[92]. This sampler consists of an electret, a small disc of polymer that carries a permanent electrical charge. It captures particles by electrical attraction, at a rate dependent on the particle’s electrical mobility and independent of wind speed. These samplers were designed for monitoring personal exposure to industrial aerosols, but exposure in urban environments suggests that they have potential for outdoor monitoring. At present, however, the samplers are only used for assessing the mass of particulate material in the atmosphere. They do not collect sufficient material for the detection of associated POPs — tens of micrograms collected per sample, only[92]. However, it may be possible to develop and adapt this technique for environmental POP monitoring.

### Wet and Dry Deposition Sampling

Wet and dry deposition can be measured together by means of a Frisbee or funnel that directs any rainwater etc. into a collecting vessel[93,94]. The Frisbee/funnel and the rainwater are then extracted to find the concentration of analyte associated with total wet and dry deposition. Without electrical means, it is not possible to distinguish between the two — e.g., by making a sampler open or closed according to whether there is precipitation.

### General Comments

International (e.g., UNEP and UNECE), national (e.g., governments), and regional (e.g., local authorities) regulatory bodies currently call for monitoring of POPs in the atmosphere[95,96]. In the future, legislation is likely to increase the demand for such monitoring. The cost and location limitations for active sampling procedures will mean that there is need for alternative passive sampling technologies. This paper has summarised research that has been carried out to date. However, undoubtedly we need to improve the existing technologies and/or design new procedures for the passive sampling and detection of POPs in the atmosphere. Much work is needed to allow the detection of particle-associated species.

One sampling technology will not suit all studies. The sampler chosen will depend on the analytes of interest and on the length of deployment required. At present, SPME and POGS perhaps hold the greatest potential for sampling more volatile POPs in short-term studies. These would be useful for immediate use following pollution incidents, for example, and they have possibilities for flux and fugacity studies. SPMDs offer a good all-around sampling technique for time-integrated deployments. Neither SPME nor SPMDs would be particularly wearer friendly, and therefore an alternative method would be needed for workplace exposure. Adaptation of diffusion tubes used for volatile organic compounds would perhaps hold possibilities for this type of work. No samplers are yet available for reliable detection of particle-associated species.

## ACKNOWLEDGEMENTS

We are grateful to the following for many stimulating discussions regarding passive sampling: Don Mackay, Trent University, Peterborough, Canada; Kees Booij, NIOZ, Texel, The Netherlands; Jim Huckins and Jim Petty, USGS, Columbia, Missouri; Vicky Burnett, Grant Northcott, and Andy Sweetman, Lancaster University, Lancaster, UK; Philipp Mayer, TNO, Delft, The Netherlands; Tom Harner, Meteorological Service of Canada, Downsview, Canada; Joop Hermens, Johannes Tolls, and Theo Sinnige, Utrecht University, Utrecht, The Netherlands. The U.K. Natural Environment Research Council, the U.K. Environment Agency, and the British Council have partially funded our research into passive air sampling techniques.
